# Joint Effect of CFH and ARMS2/HTRA1 Polymorphisms on Neovascular Age-Related Macular Degeneration in Chinese Population

**DOI:** 10.1155/2015/821918

**Published:** 2015-03-25

**Authors:** Kai Fang, Pei Gao, Jun Tian, Xueying Qin, Wenzhen Yu, Juan Li, Qing Chen, Lvzhen Huang, Dafang Chen, Yonghua Hu, Xiaoxin Li

**Affiliations:** ^1^Department of Epidemiology & Biostatistics, School of Public Health, Peking University Health Science Center, Beijing 100191, China; ^2^Department of Ophthalmology, Peking University People's Hospital, Beijing 100044, China; ^3^Key Laboratory of Vision Loss and Restoration, Ministry of Education, Beijing 100044, China; ^4^Beijing Centers of Disease Control and Prevention, Beijing 100013, China; ^5^Department of Hygiene Toxicology, Preventive Medical College, Third Military Medical University, Chongqing 400038, China

## Abstract

*Purpose*. The etiology of neovascular age-related macular degeneration (nAMD) cannot be completely explained by identified environmental risk factors or single-locus gene variants. This study was to explore the potential interactions among gene variants on nAMD in Chinese population. *Methods*. 43 SNPs located in different genes were genotyped in 932 Chinese individuals (464 nAMD patients and 468 controls). We explored the potential interactions among gene variants using generalized multifactor dimensionality reduction (GMDR) algorithm and the method to measure the departure from the additivity model. *Results*. The joint effect that involved *CFH* rs1061170 and *HTRA1* rs3793917 was shown statistically significant (*P* < 0.001) with the highest cross-validation consistency (10/10) and the best testing balanced accuracy (64.50%). In addition, based on the method to measure the departure from the additivity model, the synergy index (*S*) was 2.63 (1.09–6.38) and the attributable proportion due to interaction (AP) was 55.7% (21.4%–89.9%), which suggested that a common pathway may exist for these genes for nAMD. Those who carried CC for rs3793917 and TC/CC for rs1061170 were at the highest risk of nAMD (OR: 9.76, 95% CI: 4.65–20.51). *Conclusions*. Evidence that the joint effect that involved *CFH* and *ARMS2/HTRA1* may contribute to the risk of neovascular AMD in Chinese population was obtained.

## 1. Introduction

Age-related macular degeneration (AMD) is the leading cause of blindness in elder people in the Western population [1–4]. A pooled analysis from North America, Europe, and Australia showed that the prevalence of AMD was 4.6% in people aged less than 85 years and 13.1% in people aged 85 years or older [5]. In the United States, 54.4% of blindness in white adults older than 40 years was attributed to AMD [6]. In Chinese population, it was one of the top three leading causes for blindness and has become increasingly prevalent along with the rapid growth of the elder population [7–9]. Neovascular AMD (nAMD), a subtype of advanced AMD, is responsible for almost 90% of severe visual loss due to AMD [10]. As a complex disease, nAMD was believed to be attributed by not only environmental but also genetic risk factors. Complement H (*CFH*) and age-related maculopathy susceptibility 2 (*ARMS2*)/high-temperature requirement factor A1 (*HTRA1*) polymorphisms were shown to play important roles in causing the disease [11–15]. The independent associations between these genes and nAMD have been validated in the literatures including our study published previously [16]. However, the etiology of nAMD cannot be completely explained by the independent effects of these risk factors. Multiple factors in different pathways may interactively cause nAMD.

Gene-gene or gene-environment interactions have been reported in previous studies, such as* CFH* and* ARMS2* [17],* CFH and CETP* [18], cigarette smoking and* CFH* [19, 20] or* ARMS2* [21, 22], the body mass index (BMI) and* CFH or ARMS2* [23], the C-reactive protein (CRP) and* CFH* [24], or* ARMS2* [25]. However, most of these results cannot be externally validated. Nevertheless, attentions have been drawn in the investigation of the joint effects among genetic and environmental risk factors. Furthermore, in Chinese population, the information on the interactions of risk factors for nAMD was still very limited [18, 20]. Whether and if so how much in Chinese population, the effects of these genetic variants interact to cause nAMD was uncertain. In this study, we hypothesized that multiple factors interact together to cause nAMD. We aimed to select the factors in potential pathways using generalized multifactor dimensionality reduction (GMDR) algorithm which can identify the most informative set for evaluating the joint effects and then to also assess the evidence of these joint effects with three measures based on the departure from additivity model. Moreover, high-risk group for nAMD using the selected SNPs was created and the corresponding odds ratios (ORs) were reported.

## 2. Materials and Methods

### 2.1. Study Participants

This study was a hospital-based multicenter case-control study, led by the People's Hospital in Beijing and the Department of Epidemiology and Biostatistics in Peking University. Details of this study have been published previously [16]. Briefly, participants were recruited from 16 centers located in the northern, middle, and southern parts of China. The inclusion and exclusion criteria of this study were shown in Table 1. Each participant received comprehensive ophthalmologic examinations, including visual acuity, slit-lamp biomicroscopy, and fundoscopy. If the participant had the sign of AMD by fundoscopy, he/she would be further examined with fluorescence fundus angiography (FFA, Topcon TRC-50EX), optical coherence tomography (OCT, Zeiss-humphrey, Dublin, California, USA) or indocyanine green angiography (ICGA, Heidelberg Spectralis HRA). Patients were classified into different stages of AMD according to the criteria of the Age-Related Eye Disease Study (AREDS) [26]. Neovascular AMD was defined as being present if one of the following criteria was met: (1) choroidal neovascularization (CNV), (2) sensory retinal detachment, (3) retinal pigment epithelia (RPE) serous/hemorrhagic detachment, (4) subretinal/subRPE fibrosis, or (5) disciform scars. Visitors to the ophthalmologic department were taken as controls if they fit the inclusion and exclusion criteria. All of the diagnoses of AMD were performed by the ophthalmologists in the local hospital who had received standardized training and then reviewed by the experts in Peking University People's Hospital photograph reading center.

### 2.2. Ethics Statement

This study was approved by the Ethics Committee of Peking University People's Hospital. The procedures adhered to the Declaration of Helsinki. All participants were fully informed and the written informed consent from each participant was obtained.

### 2.3. Genotyping

Genomic DNA was extracted from the peripheral blood leukocytes with a DNA extraction kit (DP319-01; Tiangen Biotech, Beijing, China). Genotypes were detected by the Massarray Compact System (Sequenom, Inc., CA, USA). All single nucleotide polymorphisms (SNPs) had genotyping success rates of >97%. Hardy-Weinberg equilibrium for each SNP was checked with the* chi-square*-test in control group. *P* < 0.05 was considered as statistically significant. 43 SNPs in 10 genes were selected according to literatures review and genotyped originally [16]. 42 SNPs have shown no significant deviation from Hardy-Weinberg equilibrium in the control group (*P* > 0.05) whereas* CETP*rs173539 was excluded due to the failure of the test.* C3*rs1047286 and rs2230199 were also excluded because of the low frequency of minor allele (MAF < 1%). Linkage disequilibrium (LD) within each gene/chromosome was calculated with Haploview program (version 4.1). Threshold of 0.5 was used [27], that is, *r* ^2^ > 0.5 was considered as LD.

### 2.4. Statistical Analysis

For the demographic characteristics between cases and controls, the means of age between nAMD cases and controls were compared using the unpaired *t*-test and the differences of gender and cigarette smoking status were assessed using* chi-square* tests. The age and gender adjusted ORs and 95% confidence intervals (*CI*s) of each SNP for nAMD were estimated using the logistic regression. The regression analyses were performed with SPSS software (version 16.0 for windows, SPSS Inc., IL, USA).

We presented our findings on the joint effects among risk factors based on two statistical methods, that is, generalized multifactor dimensionality reduction algorithm (GMDR) and the measurements of the departure from the additivity model. The commonly used logistic regression was known to have only modest powers for distinguishing interactions [17]. It is generally believed that the biological interaction among factors (joint effect) may be different from the statistical interaction. The biological interaction (joint effect) was often referred to as the deviation from additivity instead of multiplicativity by the corresponding disease rates [28]. Therefore, the other two approaches, GMDR and the measurements of the departure from the additivity model were sought to have stronger powers to detect the potential joint effects among factors.

GMDR is a nonparametric method derived from multifactor dimensionality reduction (MDR) algorithm which emerged from the perspective of identifying biological joint actions. Unlike other methods to investigate statistical interaction which reflected the behavior over the distribution of individuals in a population, MDR based method targeted on the intrinsic property of the underlying interactive system in which the factors were embedded [29, 30]. In this study, GMDR was implemented using GMDR software beta version 0.7 (http://www.healthsystem.virginia.edu/internet/Addiction-Genomics/). For defining the potential pool of the risk factors for GMDR to select the most informative set, there were four sets for the gene* CFH* and one set for the gene* ARMS2/HTRA1* having SNPs with LD (see Supplementary Table S1 which is available online at http://dx.doi.org/10.1155/2015/821918). Within each LD set, the SNP with the largest OR was selected as the representative of the LD set it belonged to. We also included cigarette smoking status as the environmental risk factor with other genetic factors in the potential pool for the selection. Finally, 31 risk factors in the potential pool were loaded as the initial factors for GMDR, including genotypes of 30 SNPs and the smoking status. Relief filter (ReliefF) in GMDR was applied, allowing generating a smaller set of factors which provided the most information [31]. We allowed the five factors to be selected using GMDR in the main analysis. The joint effects based on two to five factors were then explored, with the adjustment of age and gender. The model shown the significance (*P* < 0.05) by the sign test and gained maximum testing balanced accuracy and/or cross-validation consistency was defined as the best. Permutation tests involved creating 2000 permuted datasets by randomizing the disease status labels were then implemented to get an empirical *P* value of these models. Sensitivity analysis was also carried out to allow ten factors to be selected in the informative set.

Furthermore, three measures based on the method of departure-from-additivity model introduced by Rothman [32] were also calculated to assess the joint effect: the synergy index (*S*), the attributable proportion due to interaction (AP), and the relative excess risk due to interaction (RERI). This method showed that the independent risk factors adhered to an additive model and then assessed the joint effect based on the departure from this additivity model. The synergy index (*S*) was estimated by the formula (RR_11_ − 1)/((RR_10_ − 1) + (RR_01_ − 1)), where RR_11_, RR_10,_ and RR_01_ were relative risk exposed to both two factors, only the first factor, and only the second factor, respectively. *S* > 1.00 indicated that, for both risk factors included, there was the presence of at least one shared pathway in the pathogenesis of the disease. In addition, if there was no interaction existed, AP and RERI should be equal to 0. The method of departure-from-additivity model required the dichotomous exposure [28]. Hence, for rs3793917 GG/GC was combined as in recessive model and TC/CC for rs1061170 was grouped as in dominant model, because the corresponding age and gender adjusted ORs with nAMD were the largest among the recessive and dominant models. Estimations of these measures were done using Andersson et al.s' procedures by Excel [28].

Finally, based on the best model selected by GMDR, the logistic regression for nAMD with the selected SNPs for the potential joint effect was used to estimate the ORs adjusted for age and gender. Due to the limitation of the number of participants with CC in SNP rs1061170, four combinations of the selected SNPs, that is, rs3793917 and rs1061170, were generated for the estimations.

## 3. Results

Overall, 464 nAMD patients and 468 controls were recruited from 2008 to 2010. The mean age of the participants in cases and controls was 68.3 (SD 8.75) and 64.9 (SD 9.27) years, respectively. There were more men in cases (63.4%) than those in controls (46.2%). Ever and current smokers in cases (46.9%) were more than those in controls (28.6%) (Table 2). 43 SNPs were originally genotyped and minor allele frequencies ranged from 0.02 to 0.49 (Supplementary Table S2). Five factors including rs3793917, rs1061170 (Y402H), rs380390, rs2736912, and cigarette smoking status, selected by GMDR, were shown as the most informative set for the potential joint effects (Table 3).

### 3.1. ORs for nAMD Association with Each Selected Risk Factor

**Figure 1:**
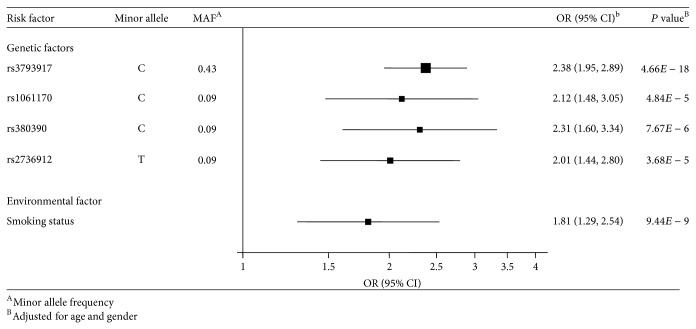
Association for nAMD with each of the five gene/environmental risk factors.

Of total 43 SNPs tested, 11 SNPs in* CFH* and 4 SNPs in* ARMS2/HTRA1* were significantly associated with nAMD. Other SNPs in* C3*,* SERPING1*,* VEGF*,* CETP*,* LPL*,* LIPC*, and* TIMP3* did not show statistically significant differences between nAMD patients and controls [16] (Supplementary Table S2). Using the additive model for each SNP, the age and gender adjusted ORs for nAMD with the selected genetic factors were 2.38 (1.95–2.89) for rs3793917, 2.12 (1.48–3.05) for rs1061170, 2.31 (1.60–3.34) for rs380390, and 2.01 (1.44–2.80) for rs2736912, respectively. The age and gender adjusted OR for the smoking status was 1.81 (1.29–2.54) (Figure 1). Similar ORs were obtained using the dominant models whereas ORs using the recessive model changed to 3.82 (2.83–5.17) for rs3793917, 7.67 (0.95–62.21) for rs1061170, 7.71 (0.95–62.31) for rs380390, and 2.11 (1.47–3.04) for rs2736912 (Supplementary Table S3).

### 3.2. Joint Effect of the Genetic Variants

**Figure 2:**
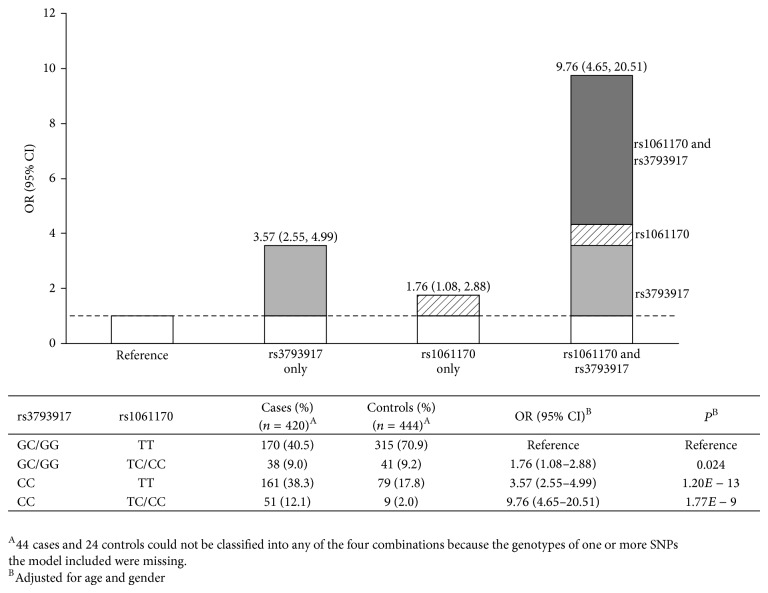
ORs with contributions from different exposure categories marked according to the departure-from-the-additivity model.

Among all the models evaluated by GMDR for the interactions, the two-factor set including rs3793917and rs1061170 has shown the highest cross-validation consistency (10/10) and the best testing balanced accuracy of 64.50% (*P* < 0.001) (Table 3). We then assessed the joint effect of rs3793917 and rs1061170 using the method of departure-from-additivity model. The synergy index (*S*) was significantly greater than 1, that is, 2.63 (1.09–6.38). AP was 55.7% (21.4%–89.9%) and RERI of the two genes was 5.44 (−1.71 to 12.59) (Table 4). For the association of the combined categories using rs3793917 and rs1061170 with nAMD, compared with the reference group, that is, GC/GG for rs3793917 and TT for rs1061170, three groups were significantly associated with nAMD after adjusting for age and gender (*P* < 0.05). The highest risk group was CC for rs3793917 and TC/CC for rs1061170 with the OR of 9.76 (4.65–20.51) (Figure 2). Sensitivity analysis was carried out to allow ten factors to be selected in the informative set. The best model remained unchanged, that is, the two-factor set including rs3793917 and rs1061170, which was shown the highest cross-validation consistency (10/10) and the best testing balanced accuracy of 64.50% (Supplementary Table S4).

## 4. Discussion

In this study, we investigated the contributions of SNPs in genes from a few different pathways, including complement system, ARMS2/HTRA1, vascular endothelial growth factor (VEGF), and high-density lipoprotein (HDL) metabolic pathway, to the risk of neovascular AMD. Independent effects of* CFH* and* ARMS2/HTRA1* genes with the risk of nAMD identified from the previous studies were confirmed. A two-locus joint effect that involved* CFH* and* ARMS2/HTRA1* genes was discovered by the GMDR analysis and by the method of departure-from-additivity model. The ORs of the categories based on the selected SNPs were then further quantified. No evidence was found in our study for the interactions of the gene variants with smoking status or factors in other tested pathways such as* CETP* in HDL metabolic pathway. Our findings, for the first time, suggested that the joint effect involved* CFH* and* ARMS2*/*HTRA1* conferred the genetic susceptibility to nAMD in Chinese population.

About the biological mechanism of CFH and ARMS2/HTRA1, previous studies suggested that CFH is a key regulator of the alternative complement pathway, involved in the pathogenesis of AMD by inflammation and innate immunity reaction [33]. Since* CFH* Y402H was reported to associate with AMD by three studies in 2005 [11–13], the wide attention has been drawn to the influence of complement system on AMD [34]. Subsequently,* ARMS2/HTRA1* on chromosome 10q26 was discovered as the second major susceptibility gene for AMD [14, 35]. However, which gene or which variant in this region was the functional element in the pathogenesis has been unclear so far. Initial studies focused on* HTRA1* because its overexpression might destroy the integrity of Bruch's membrane and lead to neovascularization [36, 37], whereas ARMS2 protein was recently found to bind several matrix proteins that had been demonstrated to cause AMD or play roles in macular dystrophies [38]. Therefore a two-way mode where upregulation of HTRA1 and downregulation of ARMS2 were supposed as the way of the 10q26 region associated with AMD [39]. Furthermore, the hypothesis about the joint effect between the complement system and* ARMS2/HTRA1* was also raised in the exploration of the mechanism of the genetic impact to nAMD. The interaction between* CFH* Y402H and* ARMS2* A69S has been detected in European population [17], and that between* CFH* I62V and* HTRA1* rs11200638 has also been reported in a Japanese study [40]. Both of these conclusions are consistent with our finding in Chinese population. The possible underlying etiology could be that* ARMS2/HTRA1* reduces the ability of the retinal pigment epithelium (RPE) to defend against oxidative stress [41] which plays a fundamental role in the pathogenesis of AMD and synergize with complement activation [42–44]. Hence, these two genes share a common pathway in systemic complement activation [45]. The possible role of* ARMS2/HTRA1* in complement activation could be a promising direction for the study about the functionality of the genes on chromosome 10q26.

Compared with the European population [17], attributable proportion due to interaction (AP) in Chinese was relatively smaller. Even though we assessed the interaction with rs800292 instead of Y402H, AP was barely changed. We also assessed the joint effect of CFH rs800292 [46] and ARMS2 rs10490924 [14, 15] with nAMD instead of the two SNPs selected by GMDR, and AP of these two genes was also very similar. This difference between European and Chinese population may be partly explained by the ethnic variation of the effect of* CFH* between Asian and European. The risk allele of* CFH* Y402H was 58.2% and OR was 5.45 for people carried at least one risk allele in European [23], whereas only 9% in our data and the corresponding OR was only around twofold. In addition, our study focused on nAMD, while the study in the European population included large drusen and geographic atrophy involving the fovea [17, 47]. Differences of inclusion criteria for AMD-related case should be taken into consideration carefully when the effect measurements were compared.

The strength and potential limitation of the current study merit consideration. It is a relatively large study in Chinese population for nAMD. The standard ophthalmologic examination for diagnosis of nAMD was implemented for all the participants. Two different statistical methods were implemented to confirm the potential joint effect. It is the first study to explore the gene-gene/environment interactions among multiple candidate factors (more than 2 factors) in Chinese population with GMDR. There are a few limitations about our study. First, although significant* CFH* ×* C2/FB* interaction was reported in AMD meta-GWAS [48], SNPs in* C2/FB* region were not genotyped in our study so that it was impossible to identify the reported interaction. Second, our study is lack of an independent dataset for validation of the findings, but cross validation in the analyses and the sensitivity analysis may make up partly. In addition, the cases enrolled in our study are not newly diagnosed ones. Given the fact of the retrospective case-control design, results involved the environmental risk factors may be biased due to confounding. Further replication studies will be helpful to reach a more convincing conclusion and interpret the inherent mechanisms.

In conclusion, the current study found a gene-gene joint effect involved* CFH* and* ARMS2/HTRA1* on neovascular AMD in Chinese population. This may help to explain the remaining susceptibility of nAMD beyond individual environmental risk factors and gene variants identified in previous studies.

## Supplementary Material

The supplementary material included the additional information about the genes and environmental risk factors selected to explore the interactions as well as the relative sensitivity analyses carried out. The full set of the genes and environmental risk factors used in GMDR for the selection of the most informative set were shown in Supplementary Table S1. Each row of the table represented a set with linkage disequilibrium or a single SNP. Within each LD set, the SNP with the largest OR was selected as the representative of the LD set it belonged to. We also included cigarette smoking status as the environmental risk factor with other genetic factors in the potential pool for the selection. Finally, 31 risk factors in the potential pool were loaded as the initial factors for GMDR, including genotypes of 30 SNPs and the smoking status.
The genotype frequency and associations of 43 SNPs with nAMD were shown in Supplementary Table S2. The minor allele frequencies ranged from 0.02 to 0.49. 11 SNPs in *CFH*, and 4 SNPs in *ARMS2/HTRA1* were significantly associated with nAMD. Other SNPs in C3, *SERPING1, VEGF, CETP, LPL, LIPC, and TIMP3* did not show statistically significant differences between nAMD patients and controls. Age and gender adjusted ORs in dominant, recessive, and additive models of the selected 4 SNPs with nAMD were shown in Supplementary Table S3.
Sensitivity analysis was carried out to allow for ten risk factors in the most informative set by GMDR. The corresponding result was shown in Supplementary Table S4. The best model remained unchanged, i.e. the two-factor set including rs3793917and rs1061170 which was shown the highest cross-validation consistency(10/10) and the best testing balanced accuracy of 64.50%.

## Figures and Tables

**Table 1:** Inclusion and exclusion criteria.

	Inclusion criteria	Exclusion criteria
nAMD patients	(i) Being of age 50 years or more (ii) Having no signs of other retina diseases (iii) Having neovascular AMD (iv) Having DNA samples for genotyping	(i) Polypoidal choroidal vasculopathy (PCV) (ii) Myopia of 6.00 diopters or above (iii) Macular nutrition disabilities (iv) Central serous chorioretinopathy (v) Any kind of vein occlusion (vi) Diabetic retinopathy (vii) Pigment phlogistic (viii) Other diseases involving malfunction of retinal photoreceptor cells

Controls	(i) Being of age 50 years or more (ii) Being confirmed negative of AMD with fundus examination (iii) Having DNA samples for genotyping	(i) Participants with too serious chronic diseases to complete questionnaire and examinations

**Table 2:** Demographic characteristics of the participants.

	nAMD case (*n* = 464)	Control (*n* = 468)	*P* value
Age (mean ± SD, years)	68.3 ± 8.75	64.9 ± 9.27	9.53*E* − 9
Gender (%)			1.31*E* − 7
Male	294 (63.4)	216 (46.2)	
Female	170 (36.6)	252 (53.8)	
Cigarette smoking (%)			9.44*E* − 9
Never	246 (53.1)	334 (71.4)	
Ever and current	217 (46.9)	134 (28.6)	

**Table 3:** Selected models for nAMD using one to five factors by GMDR.

Number of factors included	Selected factors in the best model	Testing balanced accuracy	Sign test (*P*)	Cross-validation consistency	*P* ^A^
1	rs3793917	0.6427	0.001	10/10	<0.001
2	rs3793917, rs1061170	0.6450	0.001	10/10	<0.001
3	rs3793917, rs1061170, rs380390	0.6437	0.001	10/10	<0.001
4	rs3793917, rs1061170, rs380390, rs2736912	0.6371	0.001	8/10	<0.001
5	rs3793917, rs1061170, rs380390, rs2736912, smoking status	0.6361	0.001	10/10	<0.001

^A^Empirical *P* value of permutation test.

**Table 4:** Measures from the departure-from-additivity model for the joint effect of *CFH* rs1061170 and *HTRA1* rs3793917 with nAMD.

Measures	Estimate	Lower 95% CI	Upper 95% CI
The synergy index (*S*)^A^	2.63	1.09	6.38
AP (%)^B^	55.70%	21.40%	89.90%
RERI^C^	5.44	−1.71	12.59

^A^The synergy index (*S*) was estimated by the formula (RR_11_ − 1)/((RR_10_ − 1) + (RR_01_ − 1)).

^B^AP, the attributable proportion due to interaction, was calculated using RERI/RR_11_.

^C^The relative excess risk due to interaction (RERI) was calculated by RR_11_ − RR_10_ − RR_01_ + 1.

RR_11_, RR_10_, and RR_01_ were relative risk exposed to both two factors, only the first factor, and only the second factor, respectively.
