# Supplementary material for: Joint Effect of CFH and ARMS2/HTRA1 Polymorphisms on Neovascular Age-Related Macular Degeneration in Chinese Population
Source: J Ophthalmol. 2015 Mar 25;2015:821918. doi: 10.1155/2015/821918 (PMC4389821; doi:10.1155/2015/821918)
Supplement: Supplementary file 1 — The supplementary material included the additional information about the genes and environmental risk factors selected to explore the interactions as well as the relative sensitivity analyses carried out. The full set of the genes and environmental risk factors used in GMDR for the selection of the most informative set were shown in Supplementary Table S1. Each row of the table represented a set with linkage disequilibrium or a single SNP. Within each LD set, the SNP with the largest OR was selected as the representative of the LD set it belonged to. We also included cigarette smoking status as the environmental risk factor with other genetic factors in the potential pool for the selection. Finally, 31 risk factors in the potential pool were loaded as the initial factors for GMDR, including genotypes of 30 SNPs and the smoking status. The genotype frequency and associations of 43 SNPs with nAMD were shown in Supplementary Table S2. The minor allele frequencies ranged from 0.02 to 0.49. 11 SNPs in CFH, and 4 SNPs in ARMS2/HTRA1 were significantly associated with nAMD. Other SNPs in C3, SERPING1, VEGF, CETP, LPL, LIPC, and TIMP3 did not show statistically significant differences between nAMD patients and controls. Age and gender adjusted ORs in dominant, recessive, and additive models of the selected 4 SNPs with nAMD were shown in Supplementary Table S3. Sensitivity analysis was carried out to allow for ten risk factors in the most informative set by GMDR. The corresponding result was shown in Supplementary Table S4. The best model remained unchanged, i.e. the two-factor set including rs3793917and rs1061170 which was shown the highest cross-validation consistency(10/10) and the best testing balanced accuracy of 64.50%. [file 821918.f1.pdf]

Supporting Information

Table S1.Genes and environmental risk factors to explore the interactions in GMDR.

Gene/Environmental factor	SNP ID
CFH	[rs1061170[A],rs1329424[B],rs10801555[B],rs6695321[B],rs10733086[B]]
	[rs800292[A],rs551397[B]]
	[rs2274700[A],rs3753396[B],rs1410996[B]]
	[rs10737680[A],rs12124794[B]]
	rs7524776
	rs7542235
	rs380390
	rs3753394
C3	rs11569562
	rs7257062
	rs2250656
	rs408290
	rs2230205
	rs8112351
ARMS2/HTRA1	[rs3793917[A],rs10490924[B],rs11200638[B]]
	rs2736912
CETP	rs289744
	rs9939224
	rs2303789
	rs1532625
	rs17231506
HERPUD1	rs2133783
	rs2217332
SERPING1	rs1005510
	rs2511989
VEGF	rs833069
	rs3025039
LPL	rs12678919
LIPC	rs10468017
TIMP3	rs9621532
Cigarette smoking	-

[] refers to the set with LD. Only one SNP was selected within each set.

[A]SNP selected to represent the SNP set it belonged to.

[B]Risk factors not loaded as initial markers for GMDR.

Table S2.Genotype frequency and associations of 43 SNPs with nAMD.

Gene	SNP ID	MAF	Genotype	Cases (%)	Controls (%)	P^A	$OR(95\%CI)^B$	P^B
CFH	rs551397	0.36(A)	AA	46(10.3)	75(16.4)	2.87E-5	1.00	
			AG	183(40.9)	224(49.1)		1.23(0.80-1.89)	0.341
			GG	218(48.8)	157(34.4)		2.21(1.43-3.41)	3.41E-4
	rs800292	0.36(T)	TT	47(10.6)	76(16.7)	2.02E-5	1.00	
			TC	176(39.8)	221(48.6)		1.20(0.78-1.84)	0.408
			CC	219(49.5)	158(34.7)		2.20(1.43-3.38)	3.39E-4
	rs1061170	0.09(C)	TT	331(78.8)	395(88.8)	5.39E-5	1.00	
			TC	81(19.3)	49(11.0)		2.01(1.36-2.99)	0.001
			CC	8(1.9)	1(0.2)		8.52(1.05-69.08)	0.045
	rs1329424	0.08(A)	CC	365(79.5)	412(88.8)	9.95E-5	1.00	
			CA	86(18.7)	51(11.0)		1.88(1.28-2.76)	0.001
			AA	8(1.7)	1(0.2)		7.99(0.99-64.75)	0.052
	rs3753396	0.49(A)	GG	134(29.1)	105(22.6)	0.077		
			GA	222(48.2)	239(51.5)		NS[C]	
			AA	105(22.8)	120(25.9)		NS	
	rs6695321	0.15(A)	GG	321(70.1)	343(73.9)	0.392		
			GA	126(27.5)	113(24.4)		NS	
			AA	11(2.4)	8(1.7)		NS	
	rs7524776	0.05(C)	TT	417(90.3)	416(89.8)	0.554		
			TC	42(9.1)	46(9.9)		NS	
			CC	3(0.6)	1(0.2)		NS	
	rs7542235	0.05(G)	AA	430(93.5)	403(87.0)	0.001	1.00	
			AG	30(6.5)	58(12.5)		0.51(0.32-0.81)	0.005
			GG	0(0.0)	2(0.4)		NS	
	rs10733086	0.10(A)	TT	349(75.5)	397(85.7)	7.23E-5	1.00	
			TA	105(22.7)	65(14.0)		1.80(1.27-2.56)	0.001
			AA	8(1.7)	1(0.2)		8.06(0.99-65.35)	0.051
	rs10801555	0.08(A)	GG	363(80.0)	408(88.7)	2.30E-4	1.00	
			GA	83(18.3)	51(11.1)		1.83(1.24-2.69)	0.002
			AA	8(1.8)	1(0.2)		7.96(0.98-64.53)	0.052
	rs380390	0.09(C)	GG	348(78.2)	401(89.7)	3.23E-6	1.00	
			GC	89(20.0)	45(10.1)		2.22(1.50-3.30)	7.91E-5
			CC	8(1.8)	1(0.2)		8.67(1.07-70.18)	0.043
	rs1410996	0.39(T)	TT	54(11.7)	92(19.8)	2.55E-5	1.00	
			TC	199(43.3)	224(48.3)		1.30(0.87-1.94)	0.201
			CC	207(45.0)	148(31.9)		2.23(1.48-3.35)	1.22E-4
	rs2274700	0.39(T)	TT	51(11.6)	88(20.0)	5.42E-6	1.00	
			TC	188(42.6)	216(49.0)		1.30(0.87-1.96)	0.203
			CC	202(45.8)	137(31.1)		2.39(1.57-3.63)	4.71E-5
	rs3753394	0.45(C)	CC	89(19.4)	102(22.0)	0.127		
			CT	212(46.2)	230(49.7)		NS	
			TT	158(34.4)	131(28.3)		NS	
	rs10737680	0.39(C)	CC	54(11.7)	92(19.7)	8.25E-5	1.00	
			CA	201(43.6)	223(47.6)		1.31(0.88-1.95)	0.189

			AA	206(44.7)	153(32.7)		2.162(1.44-3.25)	2.15E-4
	rs12124794	0.32(T)	TT	39(8.6)	56(12.2)	0.006	1.00	
			TA	184(40.4)	216(47.0)		1.042(0.65-1.67)	0.864
			AA	233(51.1)	188(40.9)		1.639(1.03-2.61)	0.038
C3	rs1047286	0.002(T)	CC	455(99.6)	455(99.8)	1.000		
			CT	2(0.4)	1(0.2)		NS	
	rs11569562	0.46(T)	TT	87(18.9)	101(21.8)	0.284		
			TC	250(54.3)	228(49.2)		NS	
			CC	123(26.7)	134(28.9)		NS	
	rs2230199	0.003(G)	CC	458(99.1)	463(99.6)	0.450		
			CG	4(0.9)	2(0.4)		NS	
	rs2250656	0.22(G)	AA	283(62.6)	273(59.6)	0.649		
			AG	150(33.2)	164(35.8)		NS	
			GG	19(4.2)	21(4.6)		NS	
	rs7257062	0.26(C)	TT	251(54.4)	250(53.9)	0.359		
			TC	184(39.9)	177(38.1)		NS	
			CC	26(5.6)	37(8.0)		NS	
	rs408290	0.16(G)	CC	333(72.5)	323(69.5)	0.516		
			CG	115(25.1)	127(27.3)		NS	
			GG	11(2.4)	15(3.2)		NS	
	rs2230205	0.47(A)	GG	138(29.9)	137(29.5)	0.806		
			GA	210(45.6)	221(47.5)		NS	
			AA	113(24.5)	107(23.0)		NS	
	rs8112351	0.09(T)	CC	385(83.9)	383(83.1)	0.946		
			CT	70(15.3)	74(16.1)		NS	
			TT	4(0.9)	4(0.9)		NS	
ARMS2	rs10490924	0.44(G)	GG	66(14.3)	147(31.5)	1.10E-20	1.00	
			GT	166(36.1)	223(47.8)		1.63(1.14-2.34)	0.008
			TT	228(49.6)	97(20.8)		4.85(3.31-7.12)	6.82E-16
	rs2736912	0.09(T)	TT	3(0.7)	11(2.4)	3.36E-4	1.00	
			TC	55(12.0)	91(19.8)		2.16(0.56-8.34)	0.264
			CC	402(87.4)	358(77.8)		4.30(1.15-16.01)	0.030
HTRA1	rs11200638	0.43(G)	GG	68(14.7)	140(29.9)	1.13E-18	1.00	
			GA	163(35.3)	224(47.9)		1.49(1.04-2.14)	0.031
			AA	231(50.0)	104(22.2)		4.32(2.96-6.31)	4.19E-14
	rs3793917	0.43(G)	GG	63(13.8)	141(31.1)	3.08E-22	1.00	
			GC	161(35.4)	221(48.7)		1.60(1.11-2.31)	0.012
			CC	231(50.8)	92(20.3)		5.23(3.53-7.74)	1.36E-16
CETP	rs173539	0.04(T)	CC	400(94.6)	397(95.7)	0.732		
			CT	6(1.4)	4(1.0)		NS	
			TT	17(4.0)	14(3.4)		NS	
	rs389744	0.30(C)	AA	225(50.2)	211(47.8)	0.466		
			AC	179(40.0)	193(43.8)		NS	
			CC	44(9.8)	37(8.4)		NS	
	rs2303789	0.16(G)	TT	338(73.3)	320(68.8)	0.313		
			TG	112(24.3)	131(28.2)		NS	
			GG	11(2.4)	14(3.0)		NS	

	rs9939224	0.11(T)	GG	367(79.6)	374(80.8)	0.678	
			GT	86(18.7)	84(18.1)		NS
			TT	8(1.7)	5(1.1)		NS
	rs1532625	0.29(A)	AA	39(8.5)	45(9.7)	0.305	
			GA	193(41.9)	172(37.0)		NS
			GG	229(49.7)	248(53.3)		NS
	rs17231506	0.17(T)	CC	319(69.5)	331(71.2)	0.578	
			CT	126(27.5)	116(24.9)		NS
			TT	14(3.1)	18(3.9)		NS
HERPUD1	rs2133783	0.19(G)	AA	308(66.8)	298(64.2)	0.645	
			AG	134(29.1)	148(31.9)		NS
			GG	19(4.1)	18(3.9)		NS
	rs2217332	0.09(T)	CC	382(82.9)	382(82.7)	0.997	
			CT	75(16.3)	76(16.5)		NS
			TT	4(0.9)	4(0.9)		NS
SERPING1	rs1005510	0.26(G)	AA	250(54.3)	256(55.1)	0.845	
			AG	178(38.7)	181(38.9)		NS
			GG	32(7.0)	28(6.0)		NS
	rs2511989	0.11(A)	GG	365(79.2)	371(80.0)	0.753	
			GA	86(18.7)	86(18.5)		NS
			AA	10(2.2)	7(1.5)		NS
LPL	rs12678919	0.09(G)	AA	380(82.8)	386(82.7)	0.953	
			AG	75(16.3)	76(16.3)		NS
			GG	4(0.9)	5(1.1)		NS
LIPC	rs10468017	0.16(T)	CC	325(70.7)	316(68.4)	0.746	
			CT	125(27.2)	136(29.4)		NS
			TT	10(2.2)	10(2.2)		NS
VEGF	rs833069	0.42(G)	AA	161(34.8)	155(33.3)	0.858	
			GA	222(48.1)	227(48.7)		NS
			GG	79(17.1)	84(18.0)		NS
	rs3025039	0.19(T)	CC	299(64.7)	302(65.2)	0.267	
			CT	153(33.1)	143(30.9)		NS
			TT	10(2.2)	18(3.9)		NS
TIMP3	rs9621532	0.02(C)	AA	443(96.1)	439(94.4)	0.228	
			AC	18(3.9)	26(5.6)		NS

[A]P is referred to chi-square test.

[B]Adjusted for age and gender

[C]NS is referred to not significant.

Table S3. Age and gender adjusted ORs of the selected SNPs with nAMD.

SNP	Dominant		Recessive		Additive	
	OR(95%CI)	***P***	***OR(95%CI)***	***P***	***OR(95%CI)***	***P***
rs3793917	2.68(1.91-3.77)	1.40E-8	3.82(2.83-5.17)	2.79E-18	2.38(1.95-2.89)	4.66E-18
rs1061170	2.15(1.46-3.17)	1.00E-4	7.67(0.95-62.21)	0.056	2.12(1.48-3.05)	4.84E-5
rs380390	2.37(1.61-3.49)	1.37E-5	7.71(0.95-62.31)	0.056	2.31(1.60-3.34)	7.67E-6
rs2736912	3.84(1.03-14.27)	0.044	2.11(1.47-3.04)	5.78E-5	2.01(1.44-2.80)	3.68E-5

Table S4. Selected models fornAMDby GMDR,allowingten factors in the informative set.

No. of factors included	Selected factors in the best model	Testing balanced accuracy	Sign test (***P***)	Cross-validation consistency
1	rs3793917	0.6427	0.001	10/10
2	rs3793917, rs1061170	0.6450	0.001	10/10
3	rs3793917,rs1061170, rs380390	0.6163	0.001	4/10
4	rs3793917, rs800292, rs2274700, rs11569562	0.6114	0.001	4/10
5	rs3793917, rs1061170, rs380390, rs2274700,rs11569562	0.5991	0.001	5/10
6	rs3793917, rs2274700,rs11569562,rs3753394,rs2303789, smoking status	0.5895	0.001	8/10
7	rs3793917, rs1061170, rs800292, rs2274700, rs11569562, rs2303789, smoking status	0.6108	0.001	7/10
8	rs3793917, rs1061170, rs800292, rs2274700, rs11569562, rs3753394,rs2303789, smoking status	0.6141	0.001	6/10
9	rs3793917, rs1061170, rs800292, rs2274700, rs11569562, rs3753394,rs2736912,rs2303789, smoking status	0.6235	0.001	10/10
